# Luminescence switch-on detection of protein tyrosine kinase-7 using a G-quadruplex-selective probe†
†Electronic supplementary information (ESI) available: Compound characterisation and supplementary data. See DOI: 10.1039/c5sc01320h



**DOI:** 10.1039/c5sc01320h

**Published:** 2015-05-18

**Authors:** Sheng Lin, Wei Gao, Zeru Tian, Chao Yang, Lihua Lu, Jean-Louis Mergny, Chung-Hang Leung, Dik-Lung Ma

**Affiliations:** a Department of Chemistry , Hong Kong Baptist University , Kowloon Tong , Hong Kong , China . Email: edmondma@hkbu.edu.hk; b State Key Laboratory of Quality Research in Chinese Medicine , Institute of Chinese Medical Sciences , University of Macau , Macao , China . Email: duncanleung@umac.mo; c University of Bordeaux , ARNA Laboratory , Bordeaux , France . Email: jean-louis.mergny@inserm.fr; d INSERM , U869 , IECB , Pessac , France; e Partner State Key Laboratory of Environmental and Biological Analysis , Hong Kong Baptist University , Kowloon Tong , Hong Kong , China

## Abstract

A series of luminescent iridium(iii) complexes were synthesised and evaluated for their ability to act as luminescent G-quadruplex-selective probes. The iridium(iii) complex **9** [Ir(pbi)_2_(5,5-dmbpy)]PF_6_ (where pbi = 2-phenyl-1*H*-benzo[*d*]imidazole; 5,5-dmbpy = 5,5′-dimethyl-2,2′-bipyridine) exhibited high luminescence for G-quadruplex DNA compared to dsDNA and ssDNA, and was employed to construct a G-quadruplex-based assay for protein tyrosine kinase-7 (PTK7) in aqueous solution. PTK7 is an important biomarker for a range of leukemias and solid tumors. In the presence of PTK7, the specific binding of the sgc8 aptamer sequence triggers a structural transition and releases the G-quadruplex-forming sequence. The formation of the nascent G-quadruplex structure is then detected by the G-quadruplex-selective iridium(iii) complex with an enhanced luminescent response. Moreover, the application of the assay for detecting PTK7 in cellular debris and membrane protein extract was demonstrated. To our knowledge, this is the first G-quadruplex-based assay for PTK7.

## Abstract 2

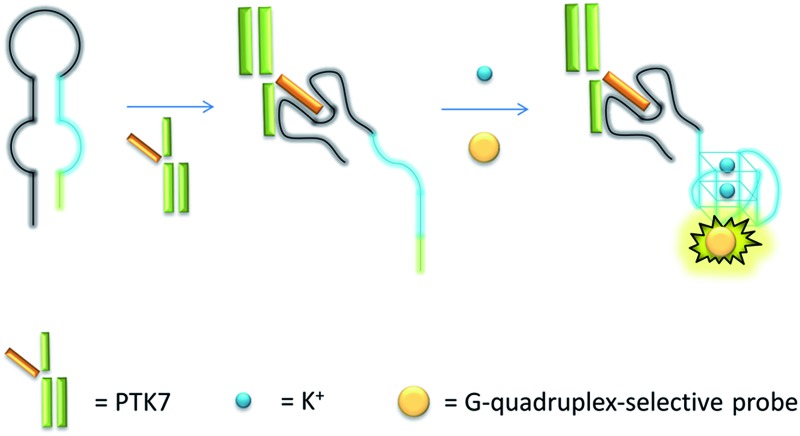
A novel luminescent G-quadruplex-selective iridium(iii) complex was employed in a G-quadruplex-based detection assay for PTK7.

## Introduction

Membrane proteins are an essential component of plasma and organellar membranes, and they play key roles in numerous physiological functions, including ion regulation, energy transduction, molecular recognition and cell communication.^1–6^ Mutations in membrane proteins have been associated with numerous human diseases.^7,8^

The cell membrane protein tyrosine kinase-7 (PTK7) is an important biomarker for a range of leukemias and solid tumors, including T-cell acute lymphoblastic leukemia (T-ALL), acute myeloid leukemia (AML), B-cell acute lymphoblastic leukemia (B-ALL), colon cancer, gastric cancer and lung cancer.^9–15^ PTK7, which is also known as colon carcinoma kinase-4 (CCK-4), contains a catalytically inactive tyrosine kinase domain.^16^ PTK7 was first identified in melanocytes,^17,18^ and was subsequently found to play a role in regulating neural development and planar cell polarity in vertebrates,^19,20^ and morphogenetic cell movement during embryonic development.^21–23^ PTK7 is also thought to be involved as a signal amplifier or modulator during cancer development and metastasis.^11,24^ PTK7 may act as a conserved modulator of multiple Wnt pathways in normal and diseased cells, including cancer cells.^23,25–27^ However, the exact functions of PTK7 in different tumors are still unclear, and a causal relationship between PTK7 overexpression and T-ALL has not yet been definitely established.^13,28^

Therefore, the effective detection of PTK7 is pivotal in order to understand the dynamic roles of this membrane protein in cells and in the pathogenesis of diseases. Currently, Western blotting, enzyme-linked immunosorbent assay (ELISA) and flow cytometry have been widely used for membrane proteins analysis.^29–31^ However, these methods tend to require multiple steps and/or sophisticated instrumentation, limiting their use for the high-throughput detection of PTK7.

Aptamers are single-stranded DNAs or RNAs that possess high affinity and binding specificity for their target molecules.^32–34^ Aptamers can be easily synthesized and modified,^35,36^ making them attractive as molecular recognition elements for the construction of sensing platforms.^37–40^ Tan and co-workers have utilized a cell-based SELEX (Systematic Evolution of Ligands by Exponential Enrichment) strategy to generate a selective aptamer sgc8, which was employed for the *in situ* detection of PTK7.^41–43^ Using sgc8, Wang *et al.* have reported an activatable aptamer probe for contrast-enhanced *in vivo* cancer imaging based on cell membrane protein-triggered alteration of conformation.^44^ The researchers have also modified the aptamer for cancer cell detection based on DNA–silver nanocluster fluorescence.^45,46^ Recently, Tang *et al.* reported a microfluidic platform for the indirect labeled detection of PTK7 by using an aptamer and nicking enzyme-assisted signal amplification.^47^

These previous studies on PTK7 detection have typically utilized the fluorescence of organic dyes or nanomaterials.^48^ However, the modification of oligonucleotides can be tedious, while the short-lived fluorescence of organic dyes may limit their application in biological tissues that exhibit a high level of background autofluorescence. On the other hand, luminescent metal complexes have attracted growing interest in optoelectronic devices,^49^ molecular imaging,^50^ chemosensing^51^ and as structural probes of biomolecules such as DNA.^52,53^ Luminescent metal complexes show selective binding affinity with specific DNA conformations and their photophysical properties can be readily modulated without the need for lengthy synthetic protocols. Additionally, most luminescent heavy metal complexes possess long emission lifetimes in the visible region and thus their phosphorescence can be readily distinguished from short-lived background auto-fluorescence. Moreover, their large Stokes shift can prevent self-quenching, while their modular synthesis allows their properties to be readily tuned without labor-intensive synthetic protocols.^49–51,54–60^ In this study, we present a luminescence switch-on detection assay for PTK7 using an iridium(iii) complex as a G-quadruplex-selective probe. This complex was identified as the top G-qudaruplex-binding probe after two successive rounds of screening of a focused library of cyclometallated iridium(iii) complexes. Based on the analysis of screening results, a brief structure–activity relationship is also presented.

The G-quadruplex is a DNA secondary structure formed from guanine-rich sequences, and consists of square-planar arrangements of guanine nucleobases stabilized by Hoogsteen hydrogen bonding and monovalent cations.^61–63^ The G-quadruplex motif has been widely used for the construction of analytical detection platforms due to its rich structural polymorphism^55,64–75^ and facile portability.^76^ To the best of our knowledge, iridium(iii) complexes have not yet been applied to the detection of PTK7 in the literature, though they have shown their potential in monitoring proteins.^52,53^

**Scheme 1:**
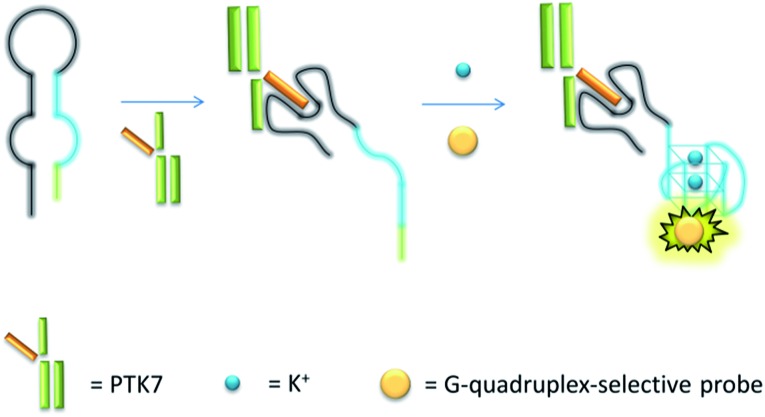
Schematic representation of the G-quadruplex-based luminescence sensing platform for PTK7 detection.

The mechanism of the proposed G-quadruplex-based assay is depicted in Scheme 1. Initially, the sgc8 aptamer sequence (black line) is partially hybridised to a G-quadruplex-forming sequence (blue line) and another short complementary sequence (green line) to form a predicted hairpin structure with two loops. In the presence of PTK7, the specific binding of the aptamer sequence would trigger a structural transition and release the G-quadruplex-forming sequence. The formation of the nascent G-quadruplex structure is then detected by the G-quadruplex-selective iridium(iii) complex with an enhanced luminescent response.

## Results and discussion

### Screening of a focus library of cyclometallated iridium(iii) complexes

**Fig. 1:**
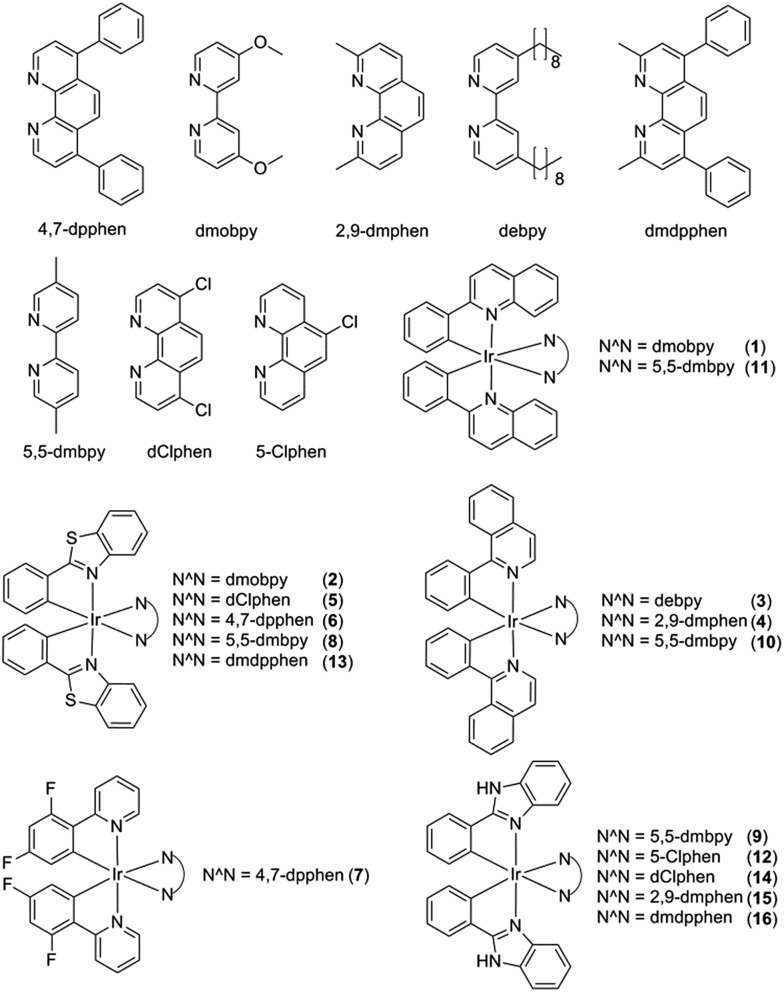
Chemical structures of cyclometallated iridium(iii) complexes **1–16**.

**Fig. 2:**
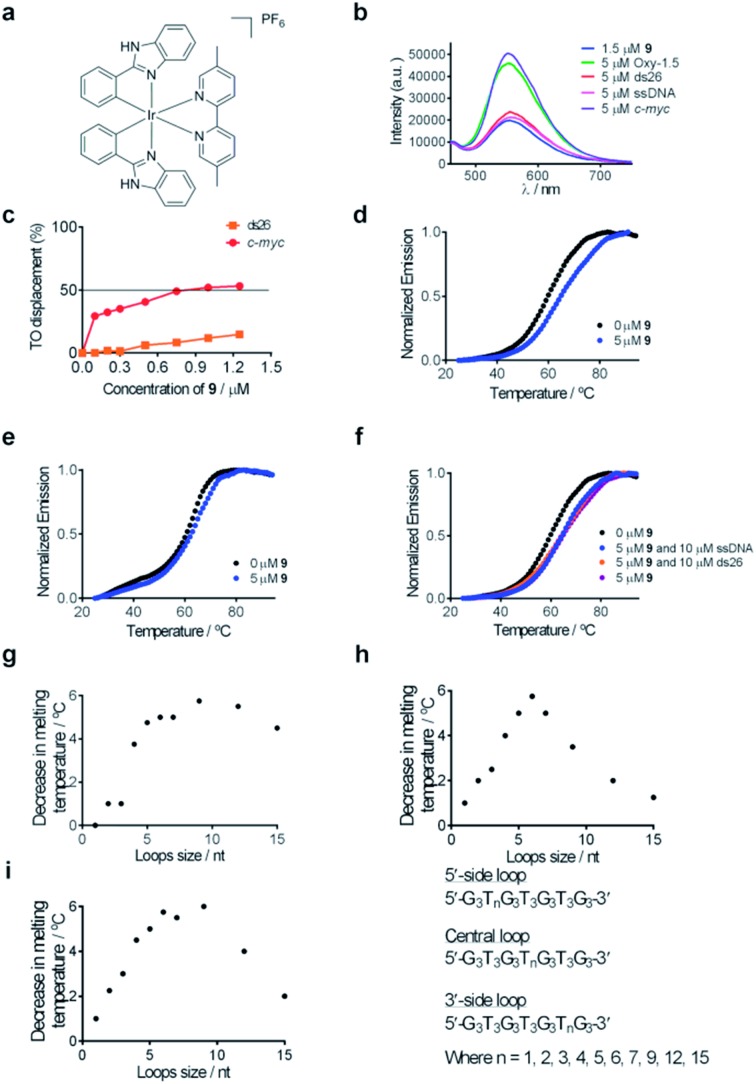
(a) Chemical structure of complex **9**. (b) Emission spectrum of complex **9** (1.5 μM) in the presence of 5 μM of ssDNA, ds26 or various G-quadruplexes. (c) G4-FID titration curves of complex **9** with duplex (ds26) or G-quadruplex (*c-myc*) DNA. (d) Melting profile of F21T G-quadruplex DNA (0.2 μM) in the absence and presence of **9** (5 μM). (e) Melting profile of F10T dsDNA (0.2 μM) in the absence and presence of **9** (5 μM). (f) Melting profile of F21T G-quadruplex DNA (0.2 μM) in the absence and presence of **9** (5 μM) and ds26 (10 μM) or ssDNA (10 μM). (g–i) Competitive FRET-melting assay results for complex **9** in the presence of G-quadruplexes with different loop lengths as the competitor. The decrease in melting temperature is shown as a function of loop size.

Initially, eight iridium(iii) complexes (**1–8**, Fig. 1) were screened for their ability to selectively distinguish *c-myc* G-quadruplex DNA from double-stranded DNA (ds26) and single-stranded DNA (ssDNA). The cyclometallated iridium(iii) complex **8**, which contains the 2-phenylbenzo[*d*]thiazole (pbtz) C^N ligand and the 5,5′-dimethyl-2,2′-bipyridine (5,5-dmbpy) N^N ligand, emerged as the top candidate, as it possessed the highest *I*_*c-myc*_/*I*_ds26_ and *I*_*c-myc*_/*I*_ssDNA_ ratios (7.5 and 5.5) out of the eight complexes tested (Fig. S1a†). Based on the structure of **8**, a focused library of eight other cyclometallated iridium(iii) complexes (**9–16**, Fig. 1) was designed and synthesised. This library was enriched in the favorable sub-structures of complex **8** that were identified in the first round of screening. Complexes **2**, **5**, **6** and **13** contain the same pbtz C^N ligand as complex **8**, but vary in their nature of the phenanthroline-based or bipyridine-based N^N ligand. Conversely, complexes **9**, **10** and **11** possess the same 5,5-dmbpy N^N ligand as **8**, but differ in their C^N ligand. In the second round of screening, the novel iridium(iii) complex **9** (Fig. 2a) which contains the 2-phenyl-1*H*-benzo[*d*]imidazole (pbi) C^N ligand and the 5,5-dmbpy N^N ligand, exhibited the greatest *I*_*c-myc*_/*I*_ds26_ and *I*_*c-myc*_/*I*_ssDNA_ ratios (Fig. S1b†).

Based on the analysis of these results, a brief structure–activity relationship can be concluded. As complexes **2**, **5**, **6**, **8** and **13** all possess the same pbtz C^N ligand, the superior *I*_*c-myc*_/*I*_ds26_ and *I*_*c-myc*_/*I*_ssDNA_ ratios of **8** could be attributed to the presence of its 5,5-dmbpy N^N ligand. This suggests that the 5,5-dmbpy ligand, which is also carried by complexes **9**, **10** and **11** in this library, might be important for G-quadruplex-binding selectivity. Additionally, despite the fact that they all possess the same pbi C^N ligand, complexes **12**, **14**, **15** and **16** all showed lower *I*_*c-myc*_/*I*_ds26_ and *I*_*c-myc*_/*I*_ssDNA_ ratios compared to complex **9**, indicating that the combination of the pbi C^N ligand and the 5,5-dmbpy N^N ligand are preferred for G-quadruplex-binding selectivity. Interestingly, the pbi ligand of complex **9** differs from the pbtz ligand of **8** by only a single atom (nitrogen instead of sulfur), suggesting that the nature of the heterocyclic core is important for the G-quadruplex affinity of these complexes.

### G-quadruplex selectivity of complex **9**

Complex **9** generated the highest luminescence response towards G-quadruplex DNA. A *ca.* 2.5-fold enhancement was observed in the luminescence signal of complex **9** at 5 μM of *c-myc* G-quadruplex DNA (Fig. 2b). On the other hand, the addition of ssDNA or dsDNA (ds17) did not induce significant changes in the luminescence intensity of **9**.

Fluorescence resonance energy transfer (FRET) melting and G-quadruplex fluorescent intercalator displacement (G4-FID) assays were employed to further validate the suitability of complex **9** as a G-quadruplex-selective probe. Although the emission region of thiazole orange (TO) (510–750 nm) and complex **9** (460–740 nm) overlap, complex **9** is not expected to interfere in the G4-FID assay due to its very low absorbance (molar extinction coefficient *ε* = 2.52 × 10^2^ dm^3^ mol^–1^ cm^–1^) at the excitation wavelength of TO (501 nm) (Fig. S2†). The results of the G4-FID assay indicated that complex **9** could displace TO from G-quadruplex structures with ^G4^DC_50_ values (half-maximal concentration of compound required to displace 50% TO from DNA) of 0.85 μM, while the displacement of TO from dsDNA was less than 15% even at the 1.25 μM of **9** tested (Fig. 2c). The selectivity of complex **9** towards G-quadruplex DNA was further investigated by FRET-melting assays. The melting temperature (Δ*T*_m_) of the F21T G-quadruplex was increased by about 6.0 °C upon the addition of 5 μM of complex **9** (Fig. 2d). By comparison, only a 2 °C increase in the melting temperature of F10T dsDNA was observed under the same conditions (Fig. 2e). Furthermore, the addition of a 50-fold higher concentration of unlabeled competitor dsDNA (ds26) or ssDNA almost did not perturb the stabilizing effect of complex **9** towards the F21T G-quadruplex (Fig. 2f). Taken together, these results indicate that complex **9** binds selectively to G-quadruplex DNA over dsDNA or ssDNA.

To examine the role of the G-quadruplex loops in the binding interaction of complex **9**, we investigated the relative binding affinity of complex **9** towards various G-quadruplex DNA structures with different loop sizes by a competitive FRET-melting assay. The G-quadruplex topologies of the sequences utilized in this experiment have been extensively validated by Mergny *et al.* ^77^ In this study, we found that the change in melting temperature was the highest at 6 or 9 nucleotides for the 5′-side loop, central loop or 3′-side loop (Fig. 2g–i). This result suggests that the G-quadruplex loop may play an important role in the G-quadruplex–**9** interaction, which is consistent with previous work by Qu and co-workers who showed that the nature of the loop region could affect the binding interaction between ligands and G-quadruplex DNA.^78^

### PTK7 detection using luminescent complex **9**

**Fig. 3:**
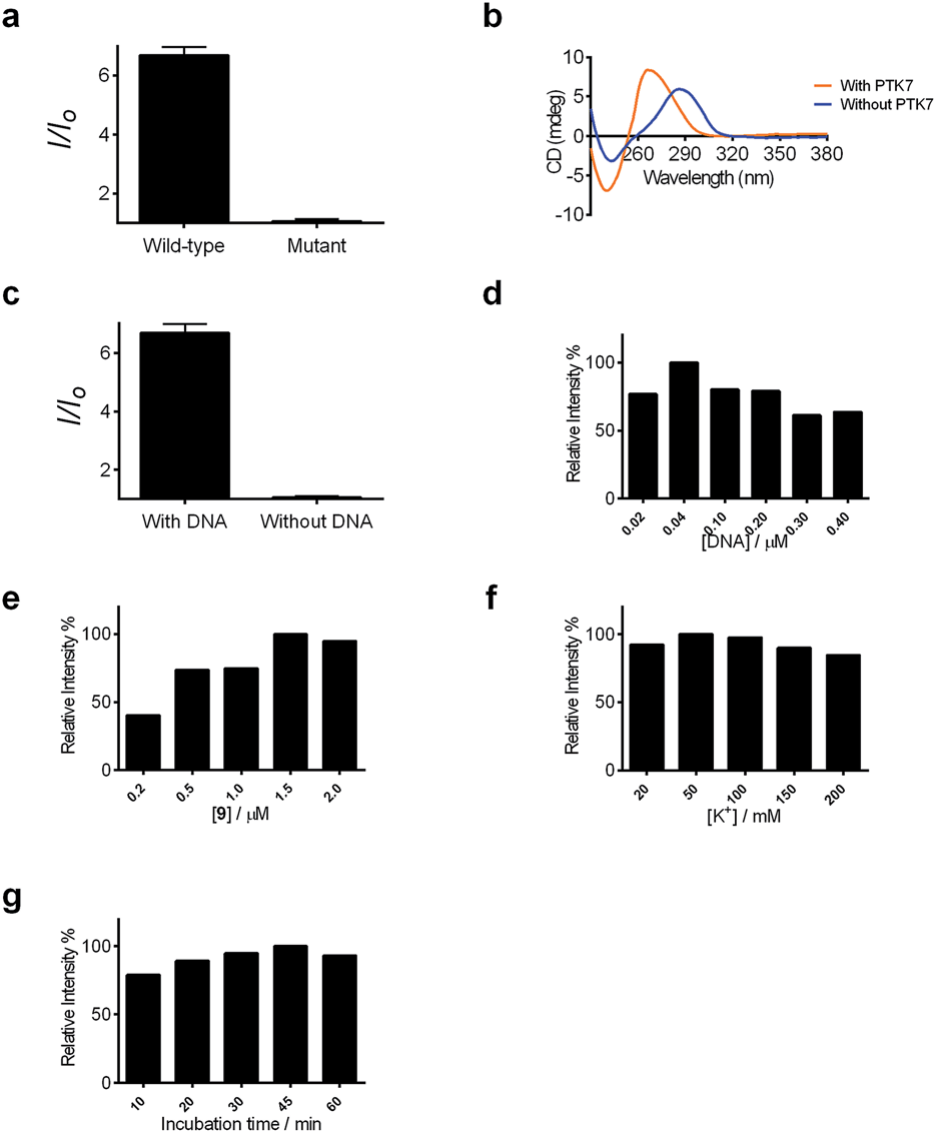
(a) Luminescence of the system in the presence of wild-type and mutant G-quadruplex DNA (0.04 μM). (b) Circular dichroism (CD) spectra with and without 18.75 nM PTK7 recorded in Tris buffer (10 mM Tris, pH 7.2). (c) Luminescence enhancement of the system in response to PTK7 (18.75 nM) in the presence or absence of hairpin DNA (0.04 μM). (d) Relative luminescence intensity of the system with different concentrations of DNA (0.02, 0.04, 0.10, 0.20, 0.30 and 0.40 μM). (e) Relative luminescence intensity of the system with different concentrations of **9** (0.2, 0.5, 1.0, 1.5 and 2.0 μM). (f) Relative luminescence intensity with different concentrations of KCl (20, 50, 100, 150 and 200 mM). (g) Relative luminescence intensity with different incubation time (10, 20, 30, 45 and 60 min). Unless otherwise stated, the concentration of complex **9** was 1.5 μM and the concentration of DNA was 0.04 μM.

Encouraged by the selective luminescence response of **9** to G-quadruplex DNA, we sought to employ **9** as a luminescent G-quadruplex probe for the detection of PTK7 as described in Scheme 1. In the absence of PTK7, complex **9** was only slightly emissive as the G-quadruplex-forming sequence remained hybridized to its partially complementary sequence. However, the luminescence signal of **9** was significantly enhanced by *ca.* 6.5-fold in the presence of PTK7 (Fig. 3a), presumably due to the alteration of the hairpin structure and the subsequent release of the G-quadruplex-forming sequence that interacted strongly with **9**. In contrast, the luminescence intensity of **9** was not significantly enhanced in response to PTK7 when a mutant G-quadruplex sequence, which lacks consecutive guanine bases and is unable to fold into a G-quadruplex, was used (Fig. 3a). This result indicates that the formation of the G-quadruplex structure plays an important role in the mechanism of PTK7 sensing platform. CD spectroscopy was also performed to validate the conformational change of DNA. In the absence of PTK7, no signal change was observed in the CD spectrum of the solution as the G-quadruplex-forming sequence remained hybridized to its complementary sequence. However, when 18.75 nM of PTK7 was added, a positive peak at 265 nm and negative peak at 240 nm appeared (Fig. 3b), indicating that the G-quadruplex-forming DNA was released from the hairpin structure and folded into a parallel G-quadruplex structure.^79^ Moreover, no significance luminescence enhancement was observed in response to PTK7 when DNA was absent, eliminating the possibility that complex **9** interacted directly with PTK7 (Fig. 3c).

### Detection sensitivity, selectivity of assay and real sample validation in biological samples

**Fig. 4:**
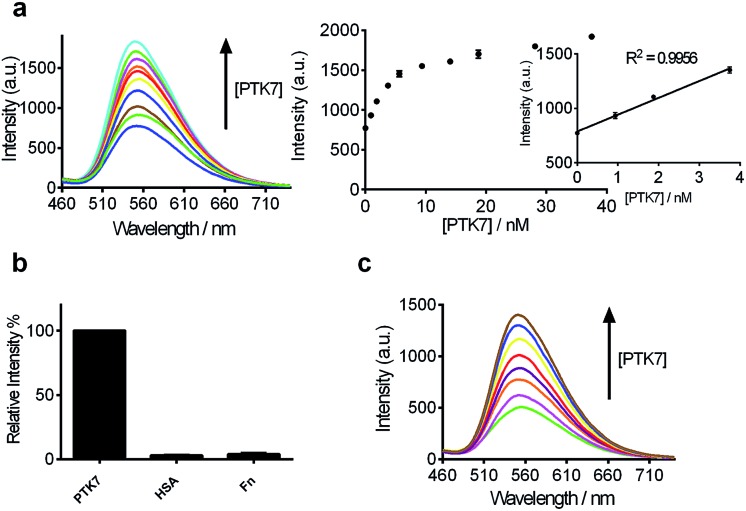
(a) Luminescence spectra and the relationship between luminescence intensity of the **9**/G4-quadruplex system at *λ* = 556 nm in response to various concentrations of PTK7: 0, 0.94, 1.88, 3.75, 5.63, 9.38, 14.06, 18.75, 28.13 and 37.50 nM. Inset: linear plot of the change in luminescence intensity at *λ* = 556 nm *vs.* PTK7 concentration. (b) Relative luminescence intensity of the system ([**9**] = 1.5 μM, [DNA] = 0.04 μM) in the presence of 18.75 nM or 5-fold excess of other proteins. (c) Luminescence spectra of the **9**/G-quadruplex system in a reaction system containing 0.5% (v/v) cell extract in response to various concentrations of PTK7: 0, 0.94, 1.88, 3.75, 5.63, 9.38, 14.06 and 18.75 nM.

After optimization of the reaction conditions such as the concentrations of KCl, complex **9** and DNA, as well as incubation time (Fig. 3d–g), we investigated the luminescence response of the system to different concentrations of PTK7. Encouragingly, the luminescence signal of **9** was enhanced as the concentration of PTK7 was increased (Fig. 4a). A detection limit of 102.8 pM was recorded using the 3*σ* method, indicating that the assay was highly sensitive for PTK7. Compared with the detection range of PTK7 and its density in living cells measured by fluorescence methods,^43,47^ the detection range of our assay might be suitable for this particular target in consideration of the *K*_d_ of sgc8 aptamer.^13,43^

The selectivity of this detection platform for PTK7 over other proteins (human serum albumin (HSA), human plasma fibronectin (Fn)) was also evaluated. The results showed that the luminescence response of the system for PTK7 was significantly stronger than that for five-fold excess concentrations of the other proteins (Fig. 4b).

To evaluate the robustness of the system, we investigated the performance of our G-quadruplex-based sensing platform for PTK7 in the presence of cellular debris (malignant melanoma A375 cells, which shows reduced expression of PTK7).^80^ In a reaction system containing 0.5% (v/v) cell extract, the **9**/G-quadruplex DNA system experienced a gradual increase in luminescence intensity as the concentration of PTK7 was increased (Fig. 4c). To verify the specificity of our detection platform for PTK7 over other membrane proteins, we extracted membrane proteins from Caco-2 cells, which show high expression of PTK7, and from K562 cells, which show no significant expression of PTK7.^14^ The luminescence of the complex **9**–DNA ensemble was enhanced in the presence of membrane proteins extracted from Caco-2 cells (Fig. S3†). On the other hand, minimal luminescence enhancement was observed with membrane proteins extracted from K562 cells, unless PTK7 was also spiked into the extract (Fig. S3b†). Furthermore, no luminescence enhancement was observed in control experiments conducted in the absence of DNA, suggesting that the structural transition of the aptamer was important for PTK7 detection in this assay. These results demonstrate that this assay could potentially be further developed for the detection of PTK7 in biological samples.

## Conclusions

In conclusion, a library of 16 luminescent iridium(iii) complexes containing various C^N and N^N ligands was screened for their ability to act as G-quadruplex probes. Iridium(iii) complex **9** was discovered to be a G-quadruplex-selective luminescent probe, and a luminescent assay for PTK7 was developed utilizing the G-quadruplex-selective property of **9**. Compared to previously reported radiographic or luminescent assays that require multiple steps and/or the use of isotopically or fluorescently labeled nucleic acids, our approach is more time and cost-effective as expensive and tedious pre-labeling or immobilization steps are avoided. Moreover, the labeling of an oligonucleotide with a fluorophore may disrupt the interaction between the oligonucleotide with its cognate target. Finally, our developed DNA-based detection platform employs luminescent transition metal complexes, which offer several advantages compared to the relatively more popular organic fluorophores, such as long phosphorescence lifetimes (>4 μs, Table S1†), large Stokes shift values and modular syntheses. Additionally, the assay could function effectively in diluted cell extract and membrane protein extract, though further optimisation may be required. We envision that our novel switch-on G-quadruplex-based luminescent detection method for PTK7 could potentially be developed as a useful tool in biochemical and biomedical research.
