# Supplementary material for: Luminescence switch-on detection of protein tyrosine kinase-7 using a G-quadruplex-selective probe
Source: Chem Sci. 2015 May 18;6(7):4284–90. doi: 10.1039/c5sc01320h (PMC5707507; doi:10.1039/c5sc01320h)
Supplement: Supplementary file 1 [file SC-006-C5SC01320H-s001.pdf]

Electronic Supplementary Material (ESI) for Chemical Science.
This journal is © The Royal Society of Chemistry 2015

Supporting Information

Luminescence switch-on detection of protein tyrosine kinase-7 using a G-quadruplex-selective probe

Sheng Lin[a], Wei Gao[a], Zeru Tian[a], Chao Yang[b], Lihua Lu[a], Jean-Louis Mergny*[cd], Chung-Hang Leung*[b] and Dik-Lung Ma*[ae]

[a] *Department of Chemistry, Hong Kong Baptist University, Kowloon Tong, Hong Kong, China. Email: edmondma@hkbu.edu.hk*

[b] *State Key Laboratory of Quality Research in Chinese Medicine, Institute of Chinese Medical Sciences, University of Macau, Macao, China. Email: duncanleung@umac.mo*

[c] *University of Bordeaux, ARNA laboratory, Bordeaux, France. Email: jean-louis.mergny@inserm.fr*

[d] *INSERM, U869, IECB, Pessac, France.*

[e] *Partner State Key Laboratory of Environmental and Biological Analysis, Hong Kong Baptist University, Kowloon Tong, Hong Kong, China.*

Experimental section

Materials

Reagents, unless specified, were purchased from Sigma Aldrich (St. Louis, MO) and used as received. Iridium chloride hydrate ($IrCl_3 \cdot xH_2O$) was purchased from Precious Metals Online (Australia). Recombinant human cell membrane protein tyrosine kinase-7 (PTK7) was purchased from Proteintech Group Inc. (USA). All oligonucleotides were synthesized by Techdragon Inc. (Hong Kong, China). DNA sequences used in this project:

ssDNA: 5'-$C_2AGT_2CGTAGTA_2C_3$-3', ds26: 5'-$CA_2TCG_2ATCGA_2T_2CGATC_2GAT_2G$-3', *c-myc*: 5'-$TGAG_3TG_3TAG_3TG_3TA_2$-3', Oxy-1.5: 5'-$G_4T_4G_4$-3', haripin DNA: 5'-$CTA_2C_2GTGAG_3TG_3TAG_3TG_3TA_3TCTA_2CTGCTGCGC_2GC_2G_3A_4TACTGTACG_2T_2AGA$-3'.

General experimental

Mass spectrometry was performed at the Mass Spectroscopy Unit at the Department of Chemistry, Hong Kong Baptist University, Hong Kong (China). Deuterated solvents for NMR purposes were obtained from Armar and used as received. ^1H and ^{13}C NMR were recorded on a Bruker Avance 400 spectrometer operating at 400 MHz (^1H) and 100 MHz (^{13}C). ^1H and ^{13}C chemical shifts were referenced internally to solvent shift (CD_3CN: ^1H, δ 1.94, 13C, δ 118.7; d_6-DMSO: 1H, δ 2.50, 13C, δ 39.5). Chemical shifts (δ) are quoted in ppm, the downfield direction being defined as positive. Uncertainties in chemical shifts are typically ±0.01 ppm for ^1H and ±0.05 for ^{13}C. Coupling constants are typically ± 0.1 Hz for ^1H-^1H and ±0.5 Hz for ^1H-^{13}C couplings. The following abbreviations are used for convenience in reporting the multiplicity of NMR resonances: s, singlet; d, doublet; t, triplet; q, quartet; m, multiplet; br, broad. All NMR data was acquired and processed using standard Bruker software (Topspin).

Photophysical measurement

Emission spectra and lifetime measurements for complexes were performed on a PTI TimeMaster C720 Spectrometer (Nitrogen laser: pulse output 337 nm) fitted with a 400 nm filter. Error limits were estimated: λ (±1 nm); τ (±10%); φ (±10%). All solvents used for the lifetime measurements were degassed using three cycles of freeze-vac-thaw. Luminescence quantum yields were determined using the method of Demas and Crosby $[Ru(bpy)_3][PF_6]_2$ in degassed acetonitrile as a standard reference solution ($\Phi_r = 0.062$) and calculated according to the following equation: $\Phi_s = \Phi_r(B_r/B_s)(n_s/n_r)^2(D_s/D_r)$ where the subscripts s and r refer to sample and reference standard solution respectively, n is the refractive index of the solvents, D is the integrated intensity, and Φ is the luminescence quantum yield. The quantity B was calculated by $B = 1 - 10^{-AL}$, where A is the absorbance at the excitation wavelength and L is the optical path length.[1]

Luminescence response of complexes towards different forms of DNA

The G-quadruplex DNA-forming sequences were annealed in Tris-HCl buffer (10 mM Tris, 20 mM KCl, pH 7.2) and were stored at –20 °C before use. Complex (1.5 μM) was added to 5 μM of ssDNA, dsDNA or G-quadruplex DNA in Tris-HCl buffer (10 mM Tris, pH 7.2).

Fluorescence resonance energy transfer (FRET) melting assay

The ability of **9** to stabilize G-quadruplex DNA was investigated using a fluorescence resonance energy transfer (FRET) melting assay. The labelled G-quadruplex-forming oligonucleotide F21T (5′-*FAM*-d($G_3[T_2AG_3]_3$)-*TAMRA*-3′; donor fluorophore *FAM*: 6-carboxyfluorescein; acceptor fluorophore *TAMRA*: 6-carboxytetramethylrhodamine) was diluted to 200 nM in a potassium cacodylate buffer (100 mM KCl, pH 7.0), and then heated to 95 °C in the presence of the indicated concentrations of **9**. The labeled duplex-forming oligonucleotide F10T (5′-FAM-dTATAGCTA-HEG-TATAGCTATAT-TAMRA-3′) (HEG linker: [($-CH_2-CH_2-O-)_6$]) was treated in the same manner, except that the buffer was changed to 10 mM lithium cacodylate (pH 7.4). Fluorescence readings were taken at intervals of 0.5 °C over the range of 25 to 95 °C.

G-quadruplex fluorescent intercalator displacement (G4-FID) assay

0.25 μM pre-folded DNA target is mixed with thiazole orange (0.50 μM for *c-myc* and 0.75 μM for ds26) in Tris-HCl buffer (10 mM, pH 7.2) containing 100 mM KCl, in a total volume of 3 mL. Each ligand addition is followed by a 3-min equilibration period after which the fluorescence spectrum is recorded. The percentage of displacement is calculated from the fluorescence area (FA, 510–750 nm, excitation, 501 nm).[2]

DNA preparation and PTK7 detection

The DNA substrate (100 μM) was dissolved in Tris-HCl buffer (10 mM, pH 7.2). The solution was heated to 95 °C for 10 min and then cooled at 0.1 °C/s to room temperature to allow the formation of the hairpin structure. The annealed product was stored at –20 °C before use. For PTK7 detection, 0.2 μL of the DNA substrate in Tris-HCl buffer (10 mM, pH 7.2) was diluted into 10 μL binding buffer (137 mM NaCl, 2.7 mM KCl, 10 mM Na_2HPO_4, 2 mM KH_2PO_4, pH 7.4) by enzyme free water with certain concentration of PTK7. After incubation at 37 °C for 45 min, the samples were diluted to 100 μL with Tris-HCl buffer (10 mM, 50 mM KCl, pH 7.2) and then incubated at room temperature for 30 min. The mixture was diluted using Tris-

HCl buffer (10 mM, pH 7.2) to a final volume of 500 μL. Finally, 1.5 μM of complex **9** was added to the mixture. Emission spectra were recorded in the 460−740 nm range using an excitation wavelength of 301 nm.

Synthesis

The complexes was prepared according to (modified) literature methods.[3] All complexes are characterized by ^{1}H-NMR, ^{13}C-NMR, high resolution mass spectrometry (HRMS) and elemental analysis. The precursor iridium(III) complex dimer $[Ir_2(C^\wedge N)_4Cl_2]$ is prepared as reported method.[4] Then, a suspension of $[Ir_2(C^\wedge N)_4Cl_2]$ (0.2 mmol) and corresponding N^N ligands (0.44 mmol) in a mixture of DCM:methanol (1:1, 20 mL) was refluxed overnight under a nitrogen atmosphere. The resulting solution was then allowed to cool to room temperature, and filtered to remove unreacted cyclometallated dimer. To the filtrate, an aqueous solution of ammonium hexafluorophosphate (excess) was added and the filtrate was reduced in volume by rotary evaoration until precipitation of the crude product occurred. The precipiate was then filtered and washed with several portions of water (2 × 50 mL) followed by diethyl ether (2 × 50 mL). The product was recrystallized by acetonitrile:diethyl ether vapor diffusion to yield the titled compound.

Complex **1**. Yield: 79%. ^{1}H NMR (400 MHz, CD_3CN) δ 8.38-8.30 (m, 4H), 8.12-8.10 (m, 2H), 7.93-7.92 (m, 2H), 7.86-7.83 (m, 2H), 7.55 (s, 2H), 7.43-7.38 (m, 4H), 7.17-7.08 (m, 4H), 6.98-6.96 (m, 2H), 6.80-6.76 (m, 2H), 6.51-6.49 (m, 2H), 3.87 (s, 6H); ^{13}C NMR (100 MHz, CD_3CN) δ 171.6, 169.0, 158.3, 152.9, 150.2, 148.9, 147.5, 141.3, 135.7, 132.3, 131.8, 130.5, 129.3, 128.7, 128.1, 126.2, 124.0, 119.2, 115.4, 116.6, 57.9; MALDI-TOF-HRMS: Calcd. for $C_{42}H_{32}IrN_4O_2$ $[M–PF_6]^+$: 817.2162, Found: 817.2135; Anal. ($C_{42}H_{32}IrN_4O_2PF_6$+$2H_2O$) C, H, N: calcd. 50.55, 3.64, 5.61, found 50.24, 3.42, 5.69.

Complex **2**. Yield: 85%. ^{1}H NMR (400 MHz, Acetone-d_6) δ 8.33 (d, J = 2.8 Hz, 2H), 8.21 (d, J = 0.4 Hz, 2H), 8.00 (d, J = 6.4 Hz, 2H), 7.97-7.95 (m, 2H), 7.49-7.45 (m, 2H), 7.33-7.31 (m, 2H), 7.27-7.22 (m, 2H), 7.13-7.09 (m, 2H), 6.91-6.87 (m, 2H), 6.50-6.44 (m, 4H), 4.07 (s, 6H); ^{13}C NMR (100 MHz, Acetone-d_6) δ 182.5, 169.3, 159.1, 152.6, 151.9, 150.3, 141.5, 134.3, 132.8, 132.6, 129.0, 127.6, 126.9, 125.0, 123.9, 118.7, 115.3, 112.2, 57.4; MALDI-TOF-HRMS: Calcd. for $C_{38}H_{28}IrN_4O_2S_2$ $[M–PF_6]^+$: 829.1283, Found: 829.1267; Anal.: ($C_{38}H_{28}IrN_4O_2S_2PF_6$+$2H_2O$) C, H, N: calcd. 45.19, 3.19, 5.55, found 45.53, 3.12, 5.52.

Complex **3**. Yield: 67% ^{1}H NMR (400 MHz, Acetone-d_6) δ 9.09 (s, 2H), 8.80 (s, 2H), 8.42(d, J = 8.0 Hz, 2H), 8.12-8.07 (m, 2H), 8.03-7.92 (m, 4H), 7.81 (s, 2H), 7.69 (s, 2H), 7.51-7.55 (m, 4H), 7.14 (s, 2H), 6.89 (t, J = 4.0 Hz, 2H), 6.37 (t, J = 8.0 Hz, 2H), 3.12 (m, 4H), 1.75-1.71 (m, 4H), 1.33-1.26 (m, 24H), 0.85 (t, J = 8.0 Hz, 6H); ^{13}C NMR (100 MHz, Acetone-d_6) δ 168.8, 156.3, 155.8, 154.2, 149.9, 145.6, 140.7, 137.1, 132.1, 131.9, 130.7, 130.5, 129.1, 128.4, 127.7, 126.7, 126.2, 124.8, 122.1, 121.9, 35.1, 31.7, 30.0, 22.4, 13.5; MALDI-TOF-HRMS: Calcd. for $C_{58}H_{64}IrN_4$ $[M–PF_6]^+$: 1009.4760, Found: 1009.4733; Anal. ($C_{58}H_{64}IrN_4IrPF_6$+H_2O) C, H, N: calcd. 59.42, 5.67, 4.78, found 59.42, 5.73, 4.89.

Complex **4**. Yield: 62%. ^{1}H NMR (400 MHz, Acetone-d_6) δ 9.11 (d, J = 8.0 Hz, 2H), 8.71 (m, J = 7.8 Hz, 2H), 8.43 (d, J = 7.8 Hz, 2H), 8.23 (s, 2H), 8.06-8.04 (m, 2H), 7.96-7.90 (m, 4H), 7.84 (d, J = 8.0 Hz, 2H), 7.76 (d, J = 8.0 Hz, 2H), 7.43 (d, J = 7.8 Hz, 2H), 7.11 (t, J = 4.0 Hz, 2H), 6.82 (t, J = 7.8 Hz, 2H), 6.45 (d, J = 7.8 Hz, 2H), 2.24 (s, 6H); ^{13}C NMR (100 MHz, Acetone-d_6) δ 168.7, 164.9, 152.2, 147.9, 144.9, 141.7, 139.1, 136.9, 131.9, 130.6, 129.8, 129.6, 128.9, 128.0, 127.5, 127.1, 126.5, 126.0, 121.9, 121.8, 121.0, 26.6; MALDI-TOF-HRMS: Calcd. for $C_{44}H_{32}IrN_4$ $[M–PF_6]^+$: 809.2256, Found: 809.2247; Anal. ($C_{44}H_{32}IrN_4PF_6$+$2H_2O$) C, H, N: calcd. 53.38, 3.67, 5.66, found 53.62, 3.52, 5.80.

Complex **5**. Yield: 82%. ^{1}H NMR (400 MHz, DMSO-d_6) δ 8.57 (s, 2H), 8.34-8.27 (m, 4H), 8.08 (d, J = 8.0 Hz, 2H), 8.00 (d, J = 7.6 Hz, 2H), 7.30 (t, J = 8.0 Hz, 2H), 7.15 (t, J = 7.2 Hz, 2H), 7.01-6.97 (m, 2H), 6.94-6.90 (m, 2H), 6.30 (d, J = 7.6 Hz, 2H), 5.81 (d, J = 8.4 Hz, 2H); ^{13}C NMR (100 MHz, DMSO-d_6) δ 181.7, 152.2, 149.3, 148.9, 147.9, 145.6, 140.5, 133.1, 132.6, 131.6, 129.6, 128.7, 127.5 126.5, 126.0, 124.9, 124.0, 117.0; MALDI-TOF-

HRMS: Calcd. for $C_{38}H_{22}Cl_2IrN_4S_2$ $[M–PF_6]^+$: 861.0292, Found: 862.0337; Anal.: ($C_{38}H_{22}Cl_2IrN_4S_2$ PF_6) C, H, N: calcd. 45.33, 2.20, 5.56, found 45.56, 2.13, 5.62.

Complex **6**. Reported[5]

Complex **7**. Reported[6]

Complex **8**. Yield: 85%. ^{1}H NMR (400 MHz, Acetone-d_6) δ 8.67 (d, J = 8.4 Hz, 2H), 8.21 (d, J = 8.2 Hz, 2H), 8.19 (d, J = 8.4 Hz, 2H), 8.05 (s, 2H), 7.97 (d, J = 7.8 Hz, 2H), 7.46 (d, J = 8.2 Hz, 2H), 7.18-7.13 (m, 4H), 6.93 (t, J = 7.4 Hz, 2H), 6.45 (d, J = 7.4 Hz, 2H), 6.35 (d, J = 8.2 Hz, 2H), 2.29 (s, 6H); ^{13}C NMR (100 MHz, Acetone-d_6) δ 181.64 , 154.44 , 150.67 , 150.64 , 149.26 , 140.56 , 140.43 , 139.36 , 133.18 , 132.00 , 131.63 , 128.09 , 126.73 , 126.07 , 124.12 , 123.72 , 123.16 , 117.69 , 17.74 ; MALDI-TOF-HRMS: Calcd. For $C_{38}H_{28}IrN_4S_2$ $[M–PF_6]^+$: 797.1377, Found: 797.1396; Anal.: ($C_{38}H_{28}IrN_4S_2$ PF_6) C, H, N: calcd. 48.45, 3.00, 5.95, found 48.53, 3.12, 5.79.

Complex **9**. Yield: 79%. ^{1}H NMR (400 MHz, Acetone-d_6) δ 8.65 (d, J = 8.4 Hz, 2H), 8.15 (d, J = 1.6 Hz, 2H), 8.11-8.09 (m, 2H), 7.88 (d, J = 7.4 Hz, 2H), 7.61 (d, J = 8.0 Hz, 2H), 7.26 (t, J = 7.2 Hz, 2H), 7.03 (t, J = 6.8 Hz, 2H), 6.95 (t, J = 7.6 Hz, 2H), 6.85 (t, J = 7.6 Hz, 2H), 6.44-6.42 (m, 2H), 5.86 (d, J = 8.0 Hz, 2H), 2.29 (s, 6H); ^{13}C NMR (100 MHz, Acetone-d_6) δ 165.6, 155.7, 152.3, 151.6, 140.7, 140.6, 139.4, 135.1, 134.2, 134.0, 131.4, 125.1, 124.6, 124.3, 124.1, 123.0, 114.5, 113.8, 18.5; MALDI-TOF-HRMS: Calcd. for $C_{38}H_{30}IrN_6$ $[M–PF_6]^+$: 763.2161, Found: 763.2167; Anal.: ($C_{38}H_{30}IrN_6PF_6$+0.5H_2O) C, H, N: calcd. 49.78, 3.41, 9.17, found 49.65, 3.53, 9.17.

Complex **10**. Yield: 58%. ^{1}H NMR (400 MHz, Acetone-d_6) δ 9. 08 (d, J = 8.0 Hz, 2H), 8.70 (d, J = 7.8 Hz, 2H), 8.42 (d, J = 7.8 Hz, 2H), 8.09-8.07 (m, 4H), 7.94-7.90 (m, 4H), 7.70 (d, J = 4.0 Hz, 4H), 7.58 (d, J = 7.8 Hz, 2H), 7.14 (t, J = 4.0 Hz, 2H), 6.91 (t, J = 8.0 Hz, 2H), 6.37 (d, J = 8.0 Hz, 2H), 2.20 (s, 6H); ^{13}C NMR (100 MHz, Acetone-d_6) δ 169.7, 154.9, 154.4, 151.3, 146.5, 141.7, 140.8, 139.8, 138.0, 133.0, 132.9, 131.6, 131.4, 130.0, 128.6, 127.7, 127.1, 124.8, 123.0, 122.8, 18.6; MALDI-TOF-HRMS: Calcd. for $C_{42}H_{32}IrN_4$ $[M–PF_6]^+$: 785.2256, Found: 785.2222; Anal. ($C_{42}H_{32}IrN_4PF_6$+1.5H_2O) C, H, N: calcd. 52.72, 3.69, 5.85, found: 52.89, 3.60, 6.00.

Complex **11**. Yield: 72%. ^{1}H NMR (400 MHz, Acetone-d_6) δ 8.55 (d, J = 1.6 Hz, 4H), 8.27 (t, J = 8.4 Hz, 4H), 8.12 (s, 2H), 7.96-7.92 (m, 4H), 7.50-7.45 (m, 4H), 7.22-7.14 (m, 4H), 6.87-6.83 (m, 2H), 6.59 (d, J = 8.0 Hz, 2H), 2.28 (s, 6H); ^{13}C NMR (100 MHz, Acetone-d_6) δ 171.2, 154.4, 152.0, 148,5, 148.4, 147.0, 141.2, 140.8, 139.4, 135.3, 132.0, 131.5, 130.1, 128.8, 128.2, 127.6, 125.9, 124.0, 123.8, 118.8, 20.1; MALDI-TOF-HRMS: Calcd. for $C_{42}H_{32}IrN_4$ $[M–PF_6]^+$: 785.2256, Found: 785.2273; Anal.: ($C_{42}H_{32}IrN_4PF_6$+0.5H_2O) C, H, N: calcd. 53.73, 3.54, 5.97, found 53.71, 3.49, 5.91.

Complex **12**. Reported[7]

Complex **13**. Yield: 71%. ^{1}H NMR (400 MHz, DMSO-d_6) δ 8.12 (d, J = 8.0 Hz, 2H), 7.89 (d, J = 7.6 Hz, 2H), 7.83 (d, J = 12.0 Hz, 4H), 7.63-7.56 (m, 6H), 7.53-7.51 (m, 4H), 7.39-7.35 (m, 2H), 7.08-7.03 (m, 4H), 6.85-6.81 (m, 2H), 6.30 (d, J = 8.0 Hz, 2H), 6.18 (d, J = 8.4 Hz, 2H), 2.05 (s, 6H); ^{13}C NMR (100 MHz, DMSO-d_6) δ 181.5, 164.8, 150.7, 149.7, 149.3, 148.9, 140.4, 135.5, 132.6, 131.8, 131.2, 130.2, 130.0, 129.6, 128.4, 128.2, 127.3, 127.1, 126.6, 124.8 124.7, 123.5, 117.6, 26.3; MALDI-TOF-HRMS: Calcd. for $C_{52}H_{36}IrN_4S_2$ $[M–PF_6]^+$: 973.2160, Found: 973.2024; Anal.: ($C_{52}H_{36}IrN_4S_2PF_6$+2H_2O) C, H, N: calcd. 54.11, 3.49, 4.85, found 54.02, 3.26, 4.91.

Complex **14**. Yield: 68%. ^{1}H NMR (400 MHz, Acetone-d_6) δ 8.54 (s, 2H), 8.39 (d, J = 5.6 Hz, 2H), 8.24 (d, J = 5.4 Hz, 2H), 7.83 (d, J = 7.6 Hz, 2H), 7.50 (d, J = 8.0 Hz, 2H), 7.12 (t, J = 7.6 Hz, 2H), 7.03 (t, J = 7.2 Hz, 2H), 6.84 (t, J = 7.6 Hz, 2H), 6.76 (t, J = 8.0 Hz, 2H), 6.24 (d, J = 7.6 Hz, 2H), 5.38 (d, J = 8.4 Hz, 2H); ^{13}C NMR (100 MHz, Acetone-d_6) δ 165.6, 165.5, 154.0, 153.4, 150.4, 150.1 149.9, 148.4, 140.6, 140.5, 138.8, 136.3, 135.3, 135.2, 134.3, 134.2, 132.4, 131.6, 131.5, 131.2, 130.1, 128.4, 128.3, 128.2 125.1, 125.0, 124.6, 124.5, 124.4, 123.4 123.3, 114.3, 114.2, 113.7; MALDI-TOF-HRMS: Calcd. for $C_{38}H_{24}Cl_2IrN_6$ $[M–PF_6]^+$: 827.1069, Found: 827.1063; Anal.: ($C_{38}H_{24}Cl_2IrN_6PF_6$) C, H, N: calcd. 46.92, 2.49, 8.64, found 47.05, 2.72, 8.82.

Complex **15**. Yield: 82%. ^{1}H NMR (400 MHz, DMSO-d_6) δ 8.71 (d, J = 4.0 Hz, 2H), 8.12 (s, 2H), 7.83 (d, J = 4.2 Hz, 2H), 7.77 (d, J = 3.8 Hz, 2H), 7.54 (d, J = 8.0 Hz, 2H), 7.14 (t, J = 8.0 Hz, 2H), 6.93 (t, J = 7.6 Hz, 2H), 6.76-6.69 (m, 4H), 6.06 (d, J = 3.6 Hz, 2H), 5.40 (d, J = 8.0 Hz, 2H), 2.10 (s, 6H); ^{13}C NMR (100 MHz, DMSO-d_6) δ 164.9, 164.2, 148.9, 148.7, 139.5, 139.0, 133.6, 133.0, 132.2, 129.6, 128.9, 127.6, 126.9, 124.1, 123.2, 123.1, 121.6, 113.0, 112.7, 27.2; MALDI-TOF-HRMS: Calcd. for $C_{40}H_{30}IrN_6$ $[M–PF_6]^+$: 787.2161, Found: 787.2192; Anal.: ($C_{40}H_{30}IrN_6PF_6$+1.5H_2O) C, H, N: calcd. 50.10, 3.47, 8.76, found 50.12, 3.64, 8.71.

Complex **16**. Yield: 77%. ^{1}H NMR (400 MHz, DMSO-d_6) δ 7.92 (s, 2H), 7.86 (s, 2H), 7.81 (d, J = 7.6 Hz, 2H), 7.60-7.57 (m, 12H), 7.19 (t, J = 7.6 Hz, 2H), 6.94 (t, J = 7.2, 2H), 6.85 (t, J = 8.0 Hz, 2H), 6.73 (t, J = 7.6 Hz, 2H), 6.10 (d, J = 7.6 Hz, 2H), 5.62 (d, J = 8.0 Hz, 2H), 2.15 (s, 6H); ^{13}C NMR (100 MHz, Acetone-d_6) δ 166.1, 165.8, 151.6, 151.5, 150.1, 141.0, 137.0, 134.8, 134.2, 133.8, 130.8, 130.6, 130.4, 130.0, 128.8, 128.3, 125.5, 125.0, 124.4, 124.4, 122.7, 114.8, 113.7, 28.4; MALDI-TOF-HRMS: Calcd. for $C_{52}H_{38}IrN_6$ $[M–PF_6]^+$: 939.2787, Found: 939.2827; Anal.: ($C_{52}H_{38}IrN_6PF_6$+H_2O) C, H, N: calcd. 56.67, 3.66, 7.63, found 56.55, 3.76, 7.69.

Table S1 Photophysical properties of iridium(III) complexes **1**–**16**.

Complex	Quantum yield	λ_{em}/ nm	Lifetime/ μs	UV/vis absorption λ_{abs} / nm (ε/ $dm^3 \cdot mol^{-1} \cdot cm^{-1}$)
**1**	0.384	560	4.829 ± 4.987 × 10^{-3}	258 (4.70 × 10^4), 332 (2.08 × 10^4), 438 (4.30 × 10^3)
**2**	0.431	526	4.826 ± 4.869 × 10^{-3}	321 (2.59 × 10^4), 414 (5.20 × 10^3)
**3**	0.098	588	4.772 ± 6.490 × 10^{-3}	289 (3.46 × 10^4), 334 (1.64 × 10^4), 438 (5.60 × 10^3)
**4**	0.071	589	4.789 ± 5.810 × 10^{-3}	271 (4.21 × 10^4), 431 (7.70 × 10^3)
**5**	0.162	585	4.667 ± 4.529 × 10^{-3}	272 (3.78 × 10^4), 311 (2.61 × 10^4)
**6**	0.243	557	4.847 ± 5.228 × 10^{-3}	282 (3.82 × 10^4)
**7**	0.385	518	4.831 ± 4.951 × 10^{-3}	224 (4.96 × 10^4)
**8**	0.485	526	4.829 ± 4.953 × 10^{-3}	251 (3.06 × 10^4), 308 (2.55 × 10^4), 406 (3.25 × 10^3)
**9**	0.511	556	4.165 ± 3.655 × 10^{-3}	300 (2.79 × 10^4)
**10**	0.111	589	4.727 ± 7.413 × 10^{-3}	289 (3.50 × 10^4), 436 (4.60 × 10^3)
**11**	0.671	558	4.539 ± 6.148 × 10^{-3}	258 (3.84 × 10^4), 438 (2.40 × 10^3)
**12**	0.092	620	2.710 ± 5.753 × 10^{-3}	274 (7.38 × 10^4), 301 (6.31 × 10^4), 372 (1.93 × 10^4)
**13**	0.554	532	4.837 ± 5.246 × 10^{-3}	284 (4.08 × 10^4)
**14**	0.150	621	4.426 ± 7.805 × 10^{-3}	263 (3.46 × 10^4), 298 (2.61 × 10^4)

**15**	0.168	557	$4.853 \pm 5.530 \times 10^{-3}$	277 (3.11×10^4), 302 (2.76×10^4), 364 (9.10×10^3)
**16**	0.207	565	$4.385 \pm 9.798 \times 10^{-3}$	288 (3.81×10^4)

Fig. S1a Diagrammatic bar array representation of the luminescence enhancement selectivity ratio of complexes **1**–**8** for *c-myc* G-quadruplex DNA over dsDNA (ds26) and ssDNA.

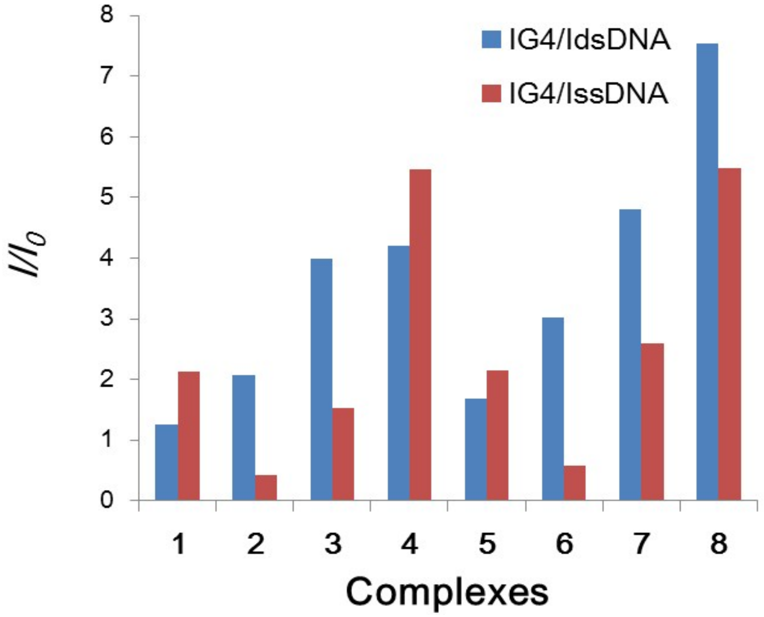

Fig. S1b Diagrammatic bar array representation of the luminescence enhancement selectivity ratio of complexes **8**–**16** for *c-myc* G-quadruplex DNA over dsDNA (ds26) and ssDNA.

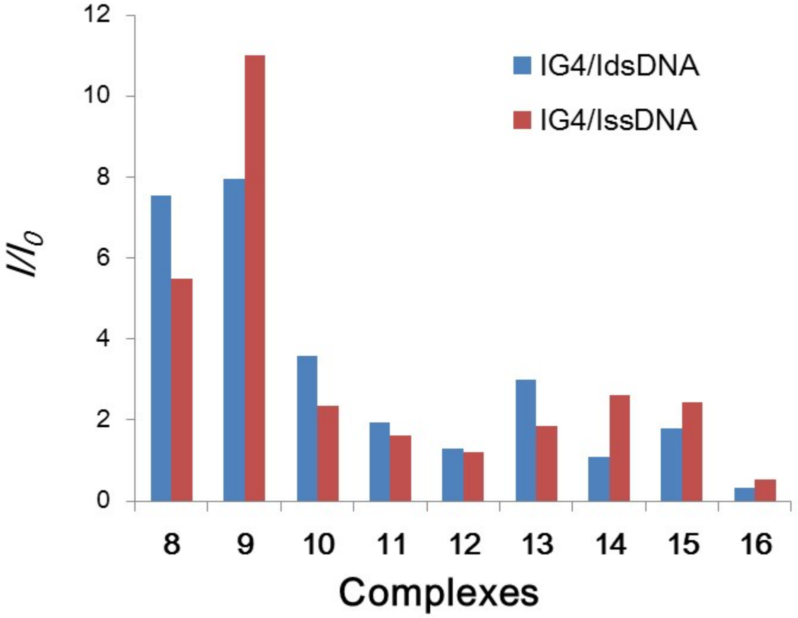

Fig. S2 UV/Vis spectrophotometric titration of complex **9** with increasing concentrations of *c-myc*.

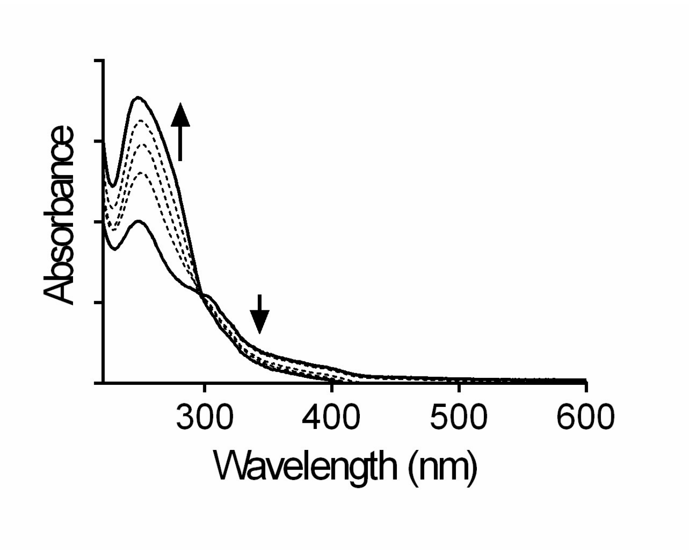

Fig. S3 Luminescence spectra of the **9**/G-quadruplex system in a reaction system containing 0.5% (v/v) membrane proteins extracted from (a) Caco-2 cells and from (b) K562 cells. The concentration of spiked PTK7 was 3.75 nM and membrane proteins were extracted from 2 × 10^5 cells. (c) Luminescence enhancement of the system in response to membrane proteins from Caco-2 cells or K562 cells in the presence of hairpin DNA (0.04 μM). (d) Luminescence spectra of the **9**/G-quadruplex system in a reaction system containing 0.5% (v/v) membrane proteins extracted from K562 cells in response to various concentrations of spiked PTK7: 0, 0.31, 0.63, 0.94, 1.88 and 3.75 nM. Membrane proteins were extracted from 2 × 10^5 cells.

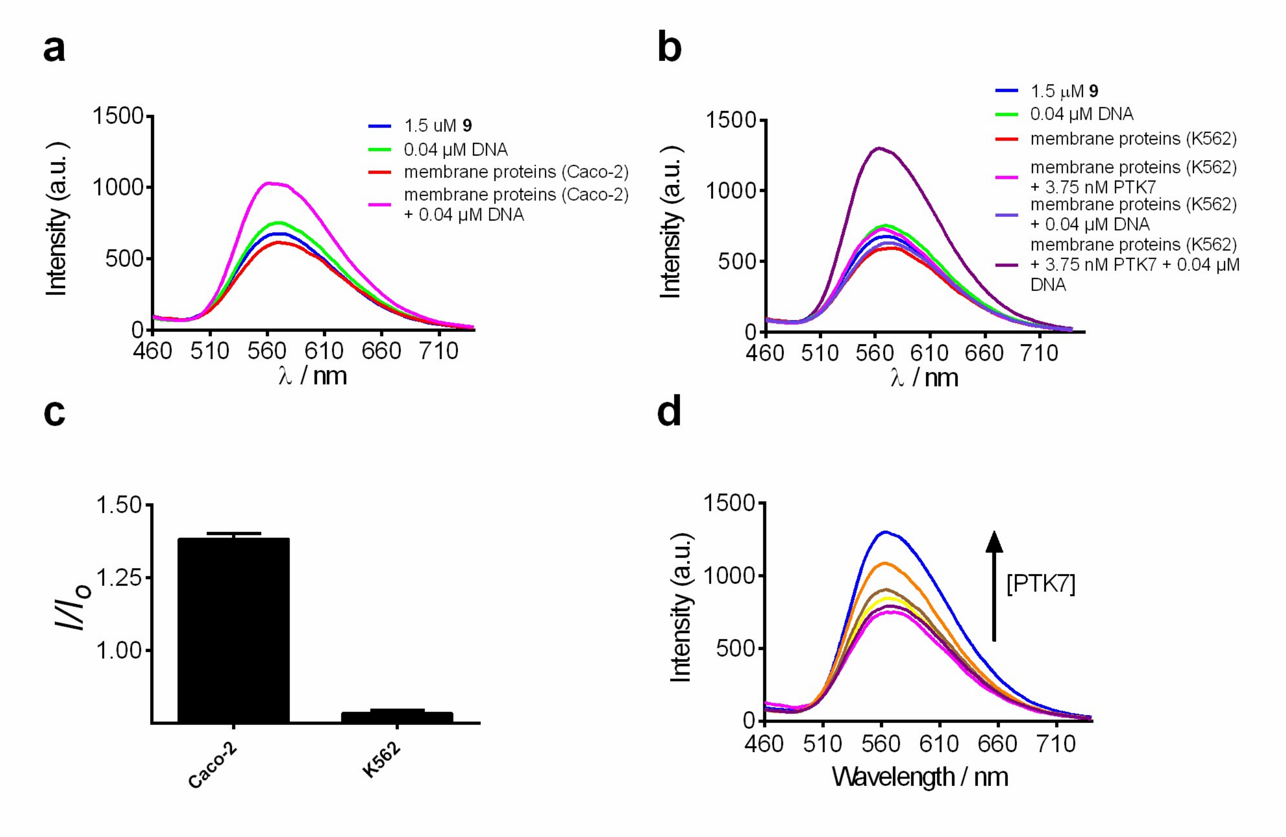

Reference

1. G. A. Crosby and J. N. Demas, *J. Phys. Chem.*, 1971, **75**, 991-1024.
2. D. Monchaud, C. Allain, H. Bertrand, N. Smargiasso, F. Rosu, V. Gabelica, A. De Cian, J. L. Mergny and M. P. Teulade-Fichou, *Biochimie*, 2008, **90**, 1207-1223.
3. D.-L. Ma, L.-J. Liu, K.-H. Leung, Y.-T. Chen, H.-J. Zhong, D. S.-H. Chan, H.-M. D. Wang and C.-H. Leung, *Angew. Chem. Int. Ed.*, 2014, **53**, 9178-9182.
4. Q. Zhao, S. Liu, M. Shi, C. Wang, M. Yu, L. Li, F. Li, T. Yi and C. Huang, *Inorg. Chem.*, 2006, **45**, 6152-6160.
5. *US Pat.*, *US20090131640-A1,* 2014.
6. S. Medina-Rodriguez, M. Marin-Suarez, J. F. Fernandez-Sanchez, A. d. l. Torre-Vega, E. Baranoff and A. Fernandez-Gutierrez, *Analyst*, 2013, **138**, 4607-4617.
7. K.-H. Leung, H.-Z. He, B. He, H.-J. Zhong, S. Lin, Y.-T. Wang, D.-L. Ma and C.-H. Leung, *Chem. Sci.*, 2015, **6**, 2166-2171.
